# Bacterial Cellulose Nanocomposites: Morphology and Mechanical Properties

**DOI:** 10.3390/ma13122849

**Published:** 2020-06-25

**Authors:** Natalia Pogorelova, Evgeniy Rogachev, Ilya Digel, Svetlana Chernigova, Dmitry Nardin

**Affiliations:** 1 Department of Food and Food Biotechnology, Omsk State Agrarian University, 644008 Omsk, Russia; na.pogorelova@omgau.org (N.P.); sv.chernigova@omgau.org (S.C.); ds.nardin@omgau.org (D.N.); 2 Department of Physics, Omsk State Technical University, 6440050 Omsk, Russia; evg.rogachev@ya.ru; 3 Institute for Bioengineering, FH Aachen University of Applied Sciences, 52428 Juelich, Germany

**Keywords:** bacterial cellulose, cellulose nanocomposites, structural characterization, SEM/EDX microscopy

## Abstract

Bacterial cellulose (BC) is a promising material for biomedical applications due to its unique properties such as high mechanical strength and biocompatibility. This article describes the microbiological synthesis, modification, and characterization of the obtained BC-nanocomposites originating from symbiotic consortium *Medusomyces gisevii*. Two BC-modifications have been obtained: BC-Ag and BC-calcium phosphate (BC-Ca_3_(PO_4_)_2_). Structure and physicochemical properties of the BC and its modifications were investigated by scanning electron microscopy (SEM), energy-dispersive X-ray spectroscopy (EDX), atomic force microscopy (AFM), and infrared Fourier spectroscopy as well as by measurements of mechanical and water holding/absorbing capacities. Topographic analysis of the surface revealed multicomponent thick fibrils (150–160 nm in diameter and about 15 µm in length) constituted by 50–60 nm nanofibrils weaved into a left-hand helix. Distinctive features of Ca-phosphate-modified BC samples were (a) the presence of 500–700 nm entanglements and (b) inclusions of Ca_3_(PO_4_)_2_ crystals. The samples impregnated with Ag nanoparticles exhibited numerous roundish inclusions, about 110 nm in diameter. The boundaries between the organic and inorganic phases were very distinct in both cases. The Ag-modified samples also showed a prominent waving pattern in the packing of nanofibrils. The obtained BC gel films possessed water-holding capacity of about 62.35 g/g. However, the dried (to a constant mass) BC-films later exhibited a low water absorption capacity (3.82 g/g). It was found that decellularized BC samples had 2.4 times larger Young’s modulus and 2.2 times greater tensile strength as compared to dehydrated native BC films. We presume that this was caused by molecular compaction of the BC structure.

## 1. Introduction

Cellulose is the most widespread natural polysaccharide, synthesized by plants, microorganisms, and some animals [1]. Due to their numerous advantageous properties, cellulose nanomaterials (CNs) have garnered a tremendous attention in engineering and medicine, as very promising components of various nanocellulose-integrated matrices, electrochromic systems, nanogenerators piezoelectric systems, etc. [2,3,4,5]. Cellulose grafting by silver nanoparticles and calcium salts has already opened many exciting possibilities. Silver nanoparticles are continuously gaining usage in antibacterial food packaging [6,7] and in medical materials, e.g., wound dressings, catheters, aprons, face masks, gloves etc. [8]. The unique bioactive properties of grafted cellulose encourage research teams around the world to focus their work on a more effective and safe cellulose-derived materials.

Many acetic acid bacteria are capable to produce extracellular cellulose matrix in static culture conditions at air/liquid boundary at 25–30 °C and pH of 4–7 [9]. A symbiotic culture of bacteria and yeast commonly called “Kombucha” is among the most effective bacterial cellulose (BC) producers. The yeast component of Kombucha mostly includes *Saccharomyces cerevisiae* and the bacterial component almost always includes *Gluconacetobacter* sp. to oxidize yeast-produced alcohols to acetic acid [10].

**Figure 1:**
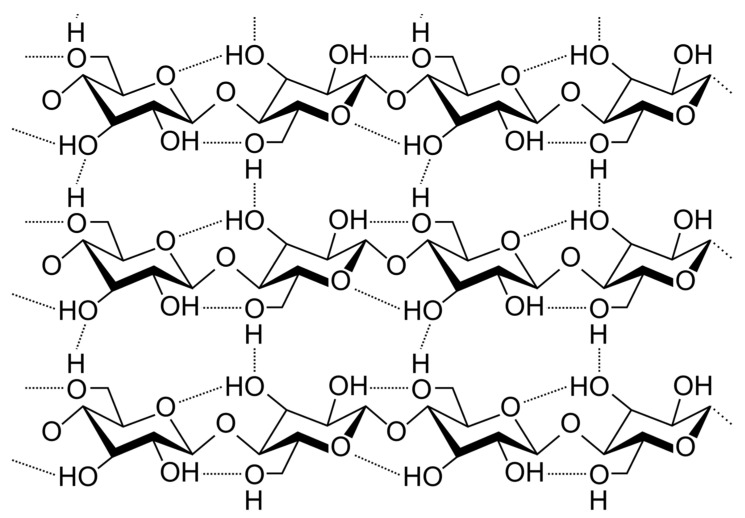
Fragment of the hypothetical bacterial cellulose structure displays extensive H-bonding, responsible for high crystallinity of this material.

Unlike plant-derived cellulose, BC has higher crystallinity (~90%) and purity, due to the absence of lignin, hemicellulose, and other components inherent in the vegetable cell wall [2,11,12]. The special properties of BC are also stipulated from peculiar orientation of glycoside chains, where numerous intramolecular and interchain hydrogen bonds give BC high strength combined with flexibility (Figure 1). Nanoscale-thick fibrils provide a high concentration of hydroxyl groups and large inner surface. As a result, a branched network is formed of BC fibrils connected to each other, in contrast to the plant cellulose, the fibers of which have a significantly greater thickness and lower density of hydrogen bonds [11,12].

Due to these aforementioned dissimilarities to plant-derived cellulose, BC has a more hydrophilic nature and higher ability to form an ultrafine web architecture, and therefore, it exhibits larger capacity for water [11]. Owing to these properties, it has been utilized for reinforcing purposes in a range of applications. In many previous works, e.g., by Field and Kerstein [13] and in a later review by J.W. Beam [14], it was shown that maintaining a moist wound environment created by an appropriate bandage, promotes healing of wounds and ulcers and reduces the pain. From this point of view, bacterial cellulose (BC) is a highly promising material. Many advantageous properties, such as high tensile strength, excellent water-holding capacity (98%), nanofibrillar structure, etc., make BC highly suitable for wound dressings. However, the lack of intrinsic antibacterial activity of BC hinders its broader use. Therefore, an additional modification/conditioning of the BC materials towards enhancing their antimicrobial activity brings many advantages [15].

The antibacterial properties of silver ions have long been known and have found a variety of applications because of broad spectrum of activity against a large number of diverse microorganisms [16,17,18]. During last decade, many approaches have been proposed, aiming to facilitate inclusion of silver ions and silver nanoparticles in various materials [16,19].

Cellulose modification by Ag-nanoparticles having defined shape and size is of great importance not only for biomedicine but also for electronics, optics, sensors, catalysis, packaging, etc. [4,20,21,22,23]. Thus, precise control over Ag-particles size and shape is important for proper functioning of many nanocomposite materials. In the published literature, there is limited information related to nanomorphological properties of the Ag-modified bacterial cellulose materials, especially for BC produced by symbiotic consortia [24,25]. Here, we provide our original data on fine morphology of silver inclusions and on the impact of silver impregnation of the BC-surface roughness.

Wound healing, being a calcium-regulated process, might also greatly benefit from an in situ Ca^2+^ supply from the dressing material. Calcium-containing nanoparticles can be designed to function as a vehicle to selectively deliver calcium to the acidic wound microenvironment, thereby accelerating wound healing [26]. It has been already reported that cellulose promotes formation of calcium salt crystals in industry and in medicine [27,28,29]. The BC with its nanofibrillar structure can be successfully used in this context for designing inorganic/organic nanocomposite materials. Although there are many promising studies devoted to BC usage as a matrix for the hydroxyapatite Ca_10_(PO_4_)_6_(OH)_2_ crystallization [30], this issue needs to be studied more thoroughly because of the complex nature of the interactions in the system “polymer matrix/hydroxyapatite crystals.” Structural and functional properties of the BC-derived materials strongly depend on their structure and composition. Probably for this reason, the reported biomechanical parameters of BC-Ca_3_(PO_4_)_2_-materials differ significantly from study to study [5,29,30]. The simultaneous structural and mechanical examination of differently modified BC-materials performed in this study may help to shed more light on the relationship between the BC-materials structure and their properties.

We studied the properties of different BC nanocomposites keeping in mind its possible future industrial production using *Medusomyces gisevii.* Since Kombucha is biotechnologically promising consortium of symbiotic yeast and *Acetobacter sp.* cells, one of the goals of this study was to investigate to what extent the structural and functional properties of the Kombucha-derived BC (including modifications) differ from the properties of the “classical” *Acetobacter*-derived cellulose materials.

Better understanding of relationships between the laboratory BC-production protocols, the obtained materials morphology (size, shape, and density) on the one hand and mechanical, water-holding, and other functional properties on the other hand, is still of great practical interest. This study presents structured comparative data concerning morphological and functional characterization of different symbiotic bacterial cellulose nanocomposites. In particular, effects of such modifications on some microstructural features, on mechanical behavior, on the water-holding properties, as well as on the surface concentration of main functional chemical groups have been examined. We also partially addressed the influence of the cultivation time (i.e., “age of the synthetized BC matrix” on the material properties.

## 2. Materials and Methods

### 2.1. Bacterial Cellulose Production and Processing of the BC Films

The BC biosynthesis was carried out by a symbiotic consortium *Medusomyces gisevii* consisting of *Gluconacetobacter sp*., *Acetobacter sp*., and *Zygosaccharomyces sp*. (as characterized by Marsh et al. [31]) under static cultivation conditions in the 250 mL Erlenmeyer flasks at 25 ± 2 °C. The consortium was isolated by the authors from traditionally fermented apple vinegar in the Omsk State Agrarian University. The culture medium consisted of 10% (w/w) glucose (Carl Roth GmbH, Karlsruhe, Germany) dissolved in 0.5% extract of plain green tea (Woodbury Sugar Shed Co. Woodbury, CT, USA). The medium was inoculated with 7-day old *Medusomyces* biomass to reach 30% (w/w) final concentration. Gel films containing BC collected after 4–13 days of cultivation were taken in the study. Each gel sample was collected in triplicate (from different flasks).

After a period of cultivation, the symbiotic culture was vigorously stirred for 10–15 min to remove the attached bacterial cells. BC films synthesized at the air/liquid boundary were separated from the culture liquid and washed with deionized water until neutral pH values (close to 7) were reached. The rinsed material was incubated in the same volume of 0.1 M NaOH at the room temperature (28 °C) for 1 h to destroy the remaining microbial cells. This step was repeated 4 times until a colorless transparent BC-film was obtained. The resulting BC film was rinsed again with the deionized water until neutral pH values (close to 7.0) were reached. Finally, BC films were dried in air at room temperature to a constant mass and stored in plastic foil at the room temperature for further tests.

### 2.2. Manufacturing and Modification of Nanocomposite BC-Films

#### 2.2.1. Modification of BC Films by Silver Nanoparticles

Spherical silver nanoparticles were prepared by sodium borohydride reduction as described in [23]. In brief, 10% glucose solution was added to the 1 mM aqueous solution of ammonium complex of silver nitrate (Carl Roth GmbH, Karlsruhe, Germany) at room temperature with constant stirring at 300 rpm. The obtained colloidal solution of silver nanoparticles was further diluted with deionized water to a concentration of 0.001 g/L. The acellular BC-gel film (area approx. 80 cm^2^) was immersed in 500 mL of the colloidal solution and shaken for 24 h, and then it was washed with plenty of distilled water for 10 min to remove the excess chemical and dried at the room temperature.

#### 2.2.2. Modification of BC Films by Calcium Phosphate

The acellular BC films, previously decellularized by NaOH as described above, were placed into 0.15 mol/L CaCl_2_ solution (pH approx. 9.0; Carl Roth GmbH, Karlsruhe, Germany) and incubated for 24 h at the room temperature. After thorough rinsing with deionized water, the BC films were immersed in 0.1 mol/L sodium phosphate (Na_3_PO_4_, Sigma-Aldrich, St. Louis, MO, USA) for next 24 h. The calcium phosphate formation within the BC-matrix was visible as increasing white opalescence. Finally, the BC samples were rinsed well with deionized water and dried at the room temperature.

### 2.3. Water-Holding Characteristics of the BC Materials

The water absorption capacity was measured as described by Thomas and Fram [32] who derived their protocol from the British Pharmacopeia for alginate dressings and packing. The hydrogel samples were cut to rectangles of 2 cm × 4 cm and weighed (initial weight W_0_). After that the samples were transferred to a centrifuge tube, the bottom of which was filled with knitted gauze to absorb the squeezed water and subjected to centrifuge forces for 15 min at 1200 rpm for dehydration. These samples were weighted again (W_1_). Finally, the weight of fully dried samples (W_4_) was measured after drying at 105 °C to a constant mass.

The swelling capacity of the samples was determined as follows: the samples were dried at 50 °C to a constant mass and then soaked for 30 min in a solution containing 2.5 mM CaCl_2_ × 2H_2_O and 142 mM NaCl (both Carl Roth GmbH, Karlsruhe, Germany) to mimic the typical ionic strength of Ca^2+^ and Na^+^ in wound exudate. After incubation, the samples were lifted with forceps for 30 s to remove the surface liquid and weighted (W_2_ value). To determine the amount of fluid held between the fibrils, swollen samples were dehydrated by centrifugation as described above and weighed (W_3_). Water-holding capacity (WHC) and water-absorption capacity (WAC) were calculated as:

WHC = (W_0_ − W_4_)/W_4_, (1)

WAC = (W_2_ − W_4_)/W_4_, (2)

Liquid held within the sample was considered as consisting of two parts:

(a) liquid held between fibers (W0 − W1 for WHC) and (W2 − W3 for WAC) and (b) liquid held inside the individual fibers (W1 − W4 for WHC) and (W3 − W4 for WAC). The corresponding W4-normalized parameters (W0 − W1)/W4; (W2 − W3)/W4; (W1 − W4)/W4; and (W3 − W4)/W4, were used to characterize and compare the dressing materials in a way similar to those applied by Qin et al. [33]. The ratios (W0 − W1)/(W1 − W4) and (W2 − W3)/(W3 − W4) reflecting the distribution of liquid in the sample of native and dried sun gel film were also evaluated. Each test was repeated 5 times. The results were presented as mean values ± standard deviations.

### 2.4. Morphological and Chemical Characterization of the BC Samples

The structural examination of the BC surface of bacterial cellulose was made by SEM/EDX microscope JEOL JCM 5700 (JEOL Ltd. Tokyo, Japan) equipped with JED-2300 Analysis Station Plus, which can perform elemental analysis by detecting characteristic X-rays generated from a specimen. The primary electron beam of the imaging system interacts with the sample and generates low-energy secondary electrons, which reflect the topographic nature of the specimen. In addition to the traditional SEM principle, the SEM/EDX technique (“energy-dispersive X-ray spectroscopy”), involves detection of characteristic X-rays, whose spectrum indicates the elemental composition of the material. In our experiments, the emitted X-rays were measured by the EDX detector to provide elemental information from the top few micrometers of the sample. For reduction of sample destruction by the primary electron beam upon observation, the measurements were conducted at 5 kV accelerating voltage. The magnifications of ×500 to ×7000 were used.

More detailed information about sample surface topology was obtained using atomic force (AFM) microscope NTEGRA Prima (NT-MDT Spectrum Instruments, Moscow, Russia) in a semicontact mode. The core of the technique is registration of the interatomic forces acting between the studied surface and the probe sensor (cantilever) fluctuating at a resonant frequency. Mechanical oscillations of the cantilever were excited by means of the piezoceramic drive. Amplitude and a phase of these fluctuations were detected by means of the power optical sensor.

The scanning was performed in the air by Si-cantilever having 5 N/m rigidity. Together with the “Height” signal, allowing to investigate the sample topography in 3 space coordinates, the “Mag” signal was registered which allowed better visualization of fine surface irregularities.

Surface roughness was calculated according to ISO 4287:1997 standard as the one-dimensional arithmetical mean deviation of the assessed profile Ra, using the formula

(3) $$R_{a}=\frac{1}{N}\overset{N}{\underset{n=1}{\sum }}(z_{n}-\bar{z})$$

where $z_{n}-\bar{z}$ represents difference between the average and measured height and N is the number of measurements.

Main functional chemical groups on the surface of the BC samples were analyzed by FTIR-spectroscopy using FTIR System Spectrum BX spectrophotometer (Perkin Elmer Co., Waltham, MA, USA) equipped with one horizontal cell (ATR Golden Gate). The FTIR spectra were obtained in the range of 4000–500 cm^−1^ with a resolution of 4 cm^−1^.

### 2.5. Mechanical Characterization of the BC Samples

Tensile tests for BC hydrogels were carried out using Instron-5543 Single-Column Universal Testing System (Instron, Melbourne, Australia). Each film was cut into three strips in the form of dumbbells (4 mm × 35 mm). The thickness of the samples was measured using a digital caliper. The two ends of the strip were fixed between the vise and moved apart at a constant speed of 1 mm/min. A 0.1 N strain gauge was used, and the force required to stretch as a function of time was recorded.

The following parameters were determined: tensile strength (σ_max_) and elongation at break (ε_max_) as the maximum stress and deformation, respectively, which the specimen could withstand until fracture. The Young’s modulus (E) was calculated from the slope of the linear region of the strain–stress curve at the tensile stage. Each test was repeated eight times, and then the mean values ± standard deviations were given.

## 3. Results and Discussion

### 3.1. Water-Holding Capacity Changes Caused by Sample Drying

The obtained pure and modified BC gel films possessed good water-holding capacity (WHC) of 62.35 ± 0.33 g/g under the physicochemical conditions close those in wounds. However, drying of the BC even at room temperatures lead to a significant decrease in its later water absorption capacity (WAC = 3.82 ± 0.12 g/g), which may be due to significant rearrangements in the internal structure of the polymer matrix induced by water loss.

BC-material represents a highly porous fibrillar network constituted by cellulose nanoribbons with sub-100 nm diameter. Therefore, water can be hold in the matrix not only by the plentiful hydroxyl groups in and on a cellulose nanofiber but also by capillary forces. The absorbed water in the samples thus can be divided into two kinds: the fraction held between the fibers defined as (W_0_ − W_1_) or (W_2_ − W_3_) and the one held inside the fibers: (W_1_ − W_4_) or (W_3_ − W_4_). The former is more inclined to migrate along textile structure, which could potentially cause macerations of surrounding healthy skin in clinical applications, while the latter is more stable, which is helpful to maintain an ideal moist healing environment [13,14]. The ratios (W_0_ − W_1_)/(W_1_ − W_4_) and (W_2_ − W_3_)/(W_3_ − W_4_) quantitatively reflect the two fractions distribution within wound dressings.

We found that the amount of water (free moisture), held between the fibrils of BC samples subjected to thermal drying represented by (W_2_ − W_3_)/W_4_ value was approximately 14 times smaller in comparison to the value (calculated as (W_0_ − W_1_)/W_4_) for the native samples, which was equal to 2.84 ± 0.06 g/g. The same but not so distinct trend was established in respect of fluid held inside fibers by absorption (bound moisture): (W_1_ − W_4_)/W_4_ was found ~2.4 times greater than (W_3_ − W_4_)/W_4_. This may indicate partial sealing of nanostructures of BC fibrils as well as the formation of new connections within and between the BC fibrils. We also found that the dried samples after swelling exhibited ~5.3 times lower free/bound moisture ratio expressed as (W_2_ − W_3_)/(W_3_ − W_4_), than the native BC samples, given by (W_0_ − W_1_)/(W_1_ − W_4_).

### 3.2. Functional Groups and the Crystallinity Degree of the BC Materials

**Figure 2:**
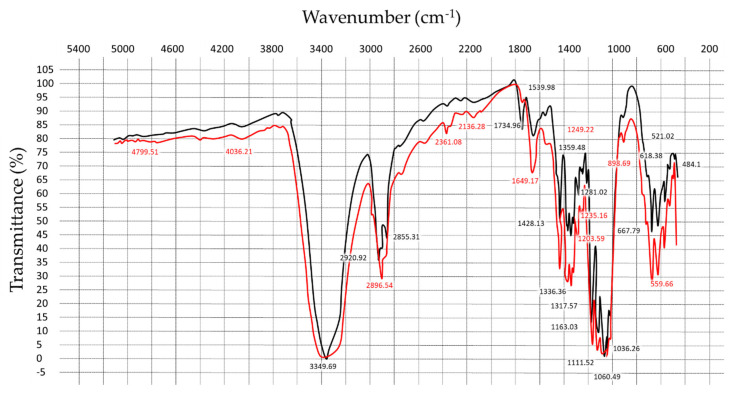
FTIR spectra of bacterial cellulose: red line—crude bacterial cellulose (BC) and black line—purified BC.

As visible from Figure 2, the native (crude) BC-samples possessed a strong characteristic peak of OH-stretching in cellulose visible at 3349.7 cm^−1^ (a wide band from 3700 to 3000 cm^−1^). Peaks at 2896.5 cm^−1^ was ascribed to the C-H stretching vibration [34]. The region 3000–2800 was related to CH_2_ and CH_2_-OH group vibrations, and the absorption band at 1649 cm^−1^ was caused by limited movements of the bound water [35]. The series of compact bands from 1200 to 1000 cm^−1^ indicated the stretching vibration of C-O-C of the ether bond as well as the stretching vibration of C-O of primary (C6) and secondary (C2, C3) alcohols.

In range of peaks visible from 1163 to 1203 cm^−1^ represented the asymmetric and symmetric stretching vibration modes of a glycoside [36]. The peak at 1428 cm^−1^ was attributed to deformation isomers and C-H_2_ bent in cellulose. The most characteristic bands for cellulose on 1036 cm^−1^ and 898 cm^−1^ peaks were connected with the stretching deformation of S-O and deformation of C1-H in a glycoside. These strips could serve as indicators of the presence of cellulose at a composite material. Thus, the obtained IR spectra of BC showed all peaks known for vegetable crystalline Avicel PH-101 cellulose, without any additional spectral features. These results confirmed that the BC samples served as raw material for nanocomposite production consisted of the purified cellulose.

Peaks at 710 cm^−1^ and 750 cm^−1^ are characteristic of Iβ cellulose and Iα cellulose, respectively. The fraction of Iα cellulose was calculated by putting the heights of peaks at 750 and 710 cm^−1^ into the equation proposed by Kataoka and Kondo [37]:

F_Iα_ = A_750_/(A_750_ + A_710_ ), (4)

where A_750_ is value of absorption at 750 cm^−1^ and A_710_ that at 710 cm^−1^. The calculated Iα fraction was close to 0.45—this was in a good agreement with the values previously reported by Keshk and Sameshima [38].

### 3.3. Microstructural Characterization of the BC Samples

Both the pure BC-films as well as BC samples modified by calcium phosphate and colloid silver were examined by scanning electron microscopy (SEM/EDX).

**Figure 3:**
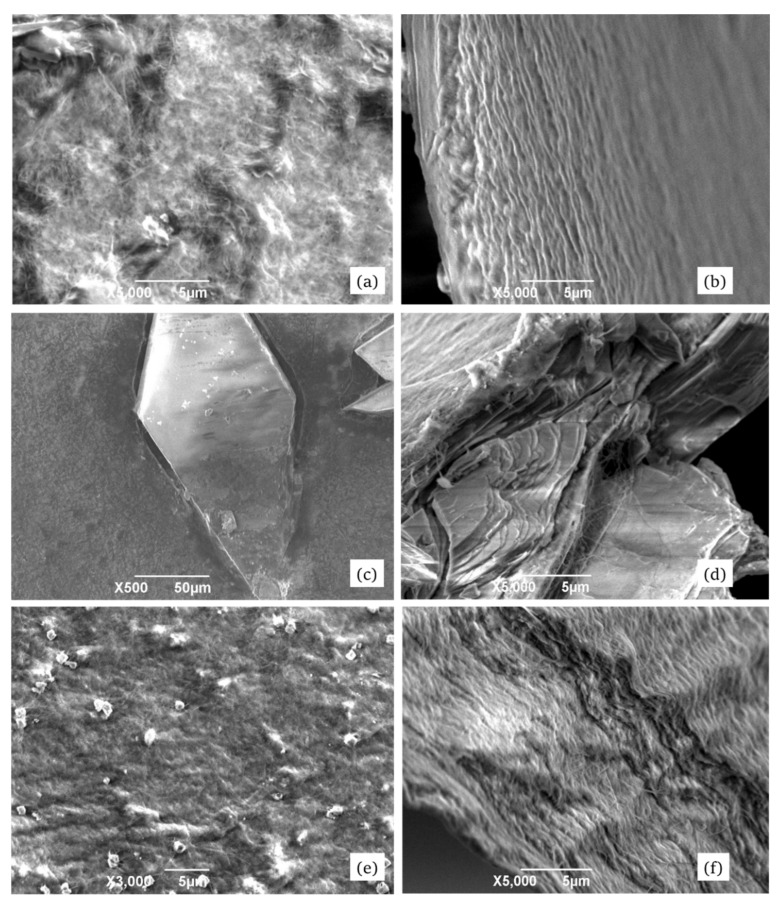
SEM images of the surface (left column of images) and cross-section (right column of images) of the pure BC film (upper row: **a** and **b**), of BC-calcium phosphate nanocomposites (in the middle: **c** and **d**), and the surface of BC film containing impregnated particles of colloid silver (lower row: **e** and **f**).

Figure 3 shows the obtained morphology data for both crude and modified BC surfaces.

As it may be visible from the Figure 3, the BC samples displayed numerous gaps between single microfibers and between their bundles. According to some literature data, bacteria excrete freshly synthetized polymer into the extracellular space. This leads to formation of an elementary fiber network which becomes more organized and stabilized by intramolecular hydrogen bonds [11,39].

Cross-sectional SEM photos of the pure BC films revealed repeating lamellar structures. The fibers of uniform size were oriented along the sample surface, having thickness of about 150 nm. The inclusions of calcium phosphate crystals (Figure 3c,d) had characteristic trigonal and hexagonal form and size of about 280 μm × 100 μm. The loner axes of the crystals were predominantly aligned along the BC surface. A distinct boundary between the organic (cellulose) and inorganic (Ca-phosphate) phases was visible. We assume that the fibrillary BC structures can serve as crystallization centers for inorganic compounds upon BC modification. The zones of inorganic inclusions displayed, in general, lower BC-fiber thickness that might cause reduction in the mechanical strength of the nanocomposite materials as compared to the pure BC films.

Figure 3e,f, shows the particles of colloid silver (having average diameter 110 nm though some reaching 0.8 µm) uniformly distributed over the surface. Interestingly, examination of the internal matrix of the samples surprisingly did not reveal visible Ag particles, even at high modifications. However, the elemental analysis confirmed their presence in the obtained nanocomposites. Another interesting micromorphological feature of the Ag-modified samples was a waving pattern in the packing of nanofibrils. Their length in some sites exceeded 15 μm, and thickness varied from 60 to 150 nm. Like in the case of Ca-phosphate modification, a distinct boundary between the main matrix material and the Ag-inclusions was visible. These boundary (contact) regions where different structures meet together might influence the mechanical strength of the materials.

**Figure 4:**
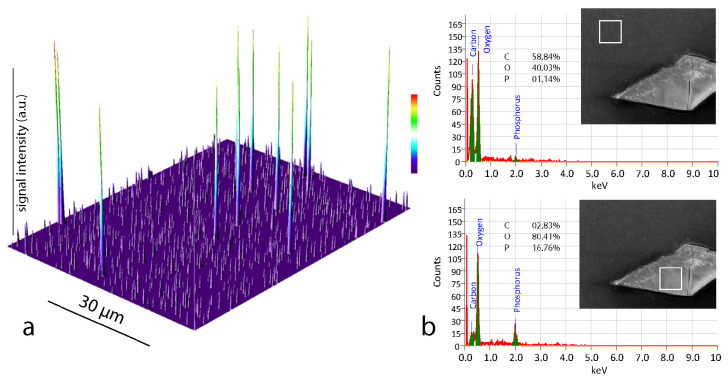
(**a**): Energy-dispersive X-ray spectroscopy (EDX) elemental mapping image of the Ag-modified BC-surface. Higher peaks represent silver atoms; numerous lower peaks are signals (shown in arbitrary units a.u.), produced by carbon atoms and (**b**) results of elemental analysis performed for BC-Ca_3_(PO_4_)_2_ surfaces in different sampling locations (indicated by white squares) with averaging over the selected area. The numbers represent the elemental composition (in %) for carbon, oxygen, and phosphorus.

The EDX analysis was used to examine the elemental composition of the BC films before and after modification. The elementary analysis of the native samples indicated carbon and oxygen content of 44.2 ± 1.6 and 49.8 ± 0.25% (mass), respectively. These data agree very well with the results for pure BC films reported previously [2,11]. The BC-Ca_3_(PO_4_)_2_ samples revealed indeed significant amount of phosphorus (as a part of the phosphoric acid residue), as visible from Figure 4. As expected, the predominant ions detected on the surface were calcium, oxygen, and phosphate. The comparison of spectrograms (Figure 4b) obtained for the main matrix (upper picture) and for the inclusion (lower picture) confirmed the elemental composition (calcium phosphate) of the crystals. Due to considerable sizes of calcium phosphate particles, the measured distribution of elements on a sample surface was very inhomogeneous.

The elemental mapping mainly was carried out in order to confirm and to visualize the presence of colloidal silver particles of the samples since precise and quantitative elemental analysis was not the subject of this article. The pseudomap shown on the Figure 4a highlights the surface distribution of Ag particles (high peaks) and C atoms (lower, “background” peaks). The presence of oxygen atoms is not shown for better visual presentation.

### 3.4. Characterization of the Samples Using AFM

Owing to the destructive action of SEM on samples at high modifications, another method, atomic force microscopy, was applied for more detailed structural examination of the surfaces.

**Figure 5:**
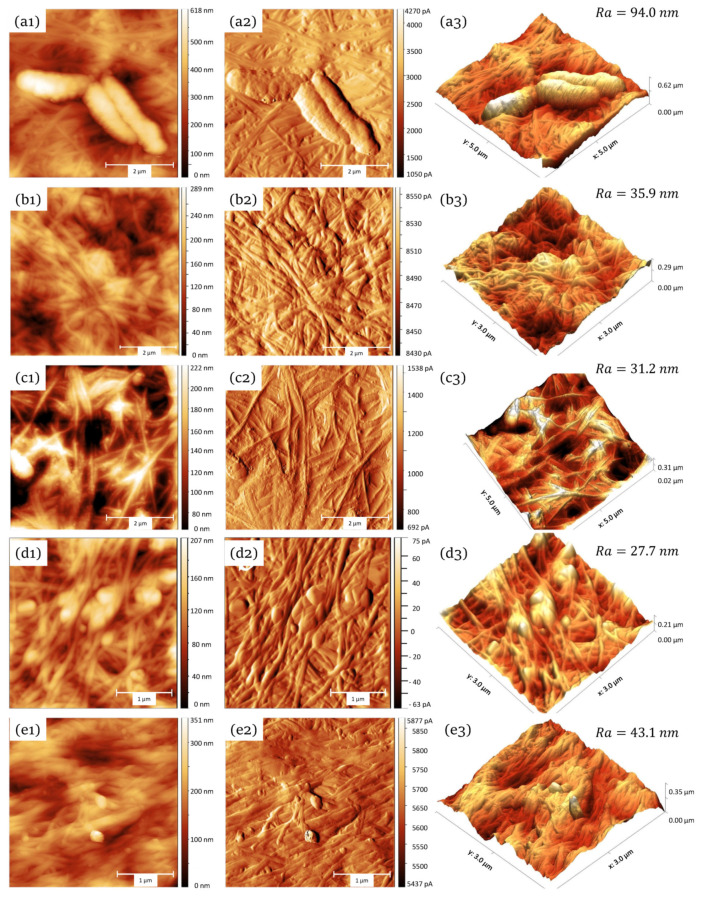
Samples surface topography obtained by atomic force microscopy (AFM): first row (**a**): crude BC with three attached bacterial cells; second row (**b**): decellularized (“purified”) BC totally free from bacterial cells; third row (**c**): younger decellularized BC matrix (4 days growth time), with higher diversity in the fibril size; forth row (**d**) BC impregnated with calcium phosphate; fifth row (**e**) BC impregnated with silver nanoparticles. The first column (1) represents the two-dimensional images of the surface. The second column (2) displays the maps of cantilever displacement amplitudes. The last column (3) shows the reconstructed three-dimensional models and the mean deviation surface roughness Ra.

The AFM microscopy delivered high-quality images of the BC-synthesizing acetic acid bacteria on the surface of the freshly prepared BC films (Figure 5a). Bacteria on the ATM images had average length of about 2 µ that matches the literature data for acetic-acid bacteria. After decellularization of the BC samples, no bacterial cells where visible, suggesting high efficacy of the applied cell removal procedure (Figure 5b). The decellularized samples displayed an entangled network of 150–165 nm thick fibrils.

The observed variability in their thickness is seemingly related to the fact that they are formed by the interlacing of thinner elementary fibrils (main structural units of BC), which takes time in the order of days. To test this hypothesis, we examined “younger” BC samples grown up within 4 days. If the assumption was correct, one could expect thinner fibrils and possibly higher diversity in their thickness. Indeed, the BC samples obtained after 4-days growth time displayed fibrils at a macrostructure forming stage. Thinner (50–60 nm) individual nanofibrils were clearly visible (Figure 5c). On the surface of nanofibrils, periodic structures having dimensions of 30–35 nm were often observed. Many fibrils exhibited twisted left-hand helices. Such geometric regularity in the microfiber forming is well known in other natural polymers, such as collagen.

Among the examined materials, the calcium phosphate-impregnated samples (Figure 5d) displayed the least surface roughness. The measured maximal height of the Ca_3_(PO_4_)_2_ reached 195 nm and the lowest profile points lied at 169 nm. Another interesting distinctive feature of such surfaces was the presence of knots consisting of entangled fibrils and calcium phosphate. The knots were apparently firmly anchored in the cellulose matrix. Although the prevailing orientation of the cellulose fibrils was random, some surface exhibited partial alignment.

The silver inclusions were difficult to find on the surface and were mostly observed in the form of roundish particles having diameter of about 110 nm, while sometimes reaching 200 nm in size (Figure 5e). The cellulose fibrils, with an average diameter of 150–160 nm, looked more aligned as compared to the Ca_3_(PO_4_)_2_-samples and displayed already mentioned waving pattern in the packing. We presume that the higher orientation order may have arisen form the stirring (at 300 rpm) applied during incubation with silver nitrate solution.

### 3.5. Mechanical Properties of the BC Samples

The materials used for wound dressing must maintain their integrity during their use. In addition, they must have the flexibility and ability to stretch, which is necessary to match the relief of the wound surface. Therefore, the measurement of mechanical characteristics (such as tensile strength) is often required to evaluate the effectiveness of materials for treating wounds [40,41].

The mechanical properties of BCs with respect to uni- and biaxial tension can be affected by both the heterogeneity of the samples thickness (due to the inhomogeneous synthesis of BCs) and the predominant orientation of fibrils. In fact, the elastic modulus of single BC-fibers was reported to be close to that of glass fiber (~78 × 10^3^ MPa) [4].

Furthermore, the strength characteristics significantly depend on the water content in BC samples [3,42]. Therefore, in the further studies, the mechanical properties of dehydrated (dried to constant weight) isotropic BC samples obtained under static cultivation conditions were analyzed before and after cleaning with a solution of sodium hydroxide. The results are presented in the Table 1 in comparison with 80 g/m^2^ office paper.

As visible form the Table 1, decellularized BC samples had 2.4 times larger Young’s modulus and 2.2 times greater tensile strength as compared to dehydrated native BC films. The native BC consists of randomly oriented parallel aggregated glucan chains in which strong covalent bonds extend in the longitudinal direction and are cross-linked by hydrogen bonds. The decellularization treatment causes significant reduction in the content of nitrogen-containing components, such as proteins and nucleic acids, as well as of low-molecular-weight polysaccharides. This, in turn, increases the likelihood of direct contacts between cellulose macromolecules. Thus, treatment with alkali results in formation of a more thermodynamically stable structure of cellulose, leading to the observed increase in the mechanical strength of the dehydrated BC samples.

Although mechanical comparison of the unmodified samples with BC-Ca_3_(PO_4_)_2_ has not revealed big differences in the measured parameters, the calcium phosphate modification seemingly increased the stiffness of the samples. The mechanical properties of the Ag-modified group were found very similar to those of the unmodified “native” samples. The same series of tests was performed on wet BC samples (hydrogels). The tested group included, together with fresh crude and decellularized wet samples, also the rehydrated samples (Table 2).

The weakening of interfibrillar bonds and the gliding and breaking of fibrils in the hydrogels of bacterial cellulose are possible due to the result of the action of water. Apparently, the network structure can provide the implementation of the so-called “stick-slip” mechanism, which consists in repeated cases of rupture and restoration of hydrogen bonds during deformation.

In a comparison of the mechanical properties of the purified cellulose samples, hydrogels before drying (not dried) and dehydrated, significantly higher (49 ± 3.6%) deformability of the fresh hydrogels compared to 18.5 ± 08% for dehydrated samples was found, while Young’s modulus in fresh samples was lower by at least two orders of magnitude (1.5 ± 0.3 vs. 250.4 ± 20.1 MPa). The found values for tensile strength and elastic modulus for wet fresh samples were comparable to those reported for the pig carotid artery (tensile strength 1.0 ± 0.2 MPa, Young’s modulus 2.3 ± 0.1 MPa), which makes it possible in the future to consider it as a framework for tissue engineering [43].

Nanocrystalline inorganic inclusions in cellulose matrix represent a great interest in medicine and industry due to their easy production and versatile functional properties. Kalantari and coworkers emphasized that Ag-containing wound dressing materials have potential for revolutionizing wound healing [44]. Similarly, wound-healing performance of cellulose bandages greatly benefits from phosphorylation, which improves the drainage efficiency, and from calcium ions that mediate the healing effect [45]. To produce a safe and effective combination of wound-healing and antibacterial properties, fabrication technologies, properties, and behavior of the grafted cellulose materials must be further thoroughly studied and optimized. We hope that the presented results will contribute to better understanding of the micromorphological and functional changes of BC-materials caused by different modification methods.

## 4. Conclusions

- In this study, we produced wet and dry bacterial cellulose (BC) films produced by Kombucha and obtained BC derivatives by (a) silver nanoparticles and (b) calcium phosphate impregnation.
- In general, we found that the structural and functional properties of the Kombucha-derived BC (including its modifications) do not differ significantly from the previously reported mechanical, water-holding, and other properties of the “classical” *Acetobacter*-derived BC materials.
- The structure of the obtained BC films was examined by SEM/EDX, AFM, and FTIR-spectroscopy, together with measurements of their mechanical and water-holding properties.
- Fine structure of the samples revealed typical multicomponent 150–160 nm thick mature fibrils of bacterial cellulose, constituted by 50–60 nm nanofibrils weaved into a left-hand helix. In younger, less mature BC samples, the thinner elements prevailed and an intermittent 30–35 nm pattern corresponding to the periodically placed links between the biopolymer macromolecules was observed on the nanofibrils surface.
- Distinctive feature of Ca-phosphate-modified BC samples were (a) in general, more chaotic structure due to the presence of entanglements and knots of 500–700 nm thick fibrils as well (b) separated inclusions of calcium phosphate crystals. The boundaries between the organic and inorganic phases were very distinct that may result in mechanical weakening of the modified samples.
- The samples impregnated with silver nanoparticles, exhibited numerous Ag inclusions on the surface in the form of roundish particles with average size of about 110 nm. Some larger particles, 190–200 nm in diameter, were observed as well. Like in the case of Ca-phosphate modification, a distinct boundary between the main matrix material and the Ag-inclusions was visible. Another interesting micromorphological feature of the Ag-modified samples was a waving pattern in the packing of nanofibrils. Their length in some sites exceeded 15 μm and thickness varied from 60 to 150 nm.
- The obtained pure and modified BC gel films possessed water-holding capacity (WHC) of 62.35 ± 0.33 g/g. However, the dried (to a constant mass) BC-films later exhibited a low water absorption capacity (WAC = 3.82 ± 0.12 g/g), which may be due to significant rearrangements in the internal structure of the polymer matrix induced by water loss.
- It was found that alkaline treatment used for decellularization of the samples lead to mechanical strengthening of the BC-matrix. Decellularized BC samples had 2.4 times larger Young’s modulus and 2.2 times greater tensile strength as compared to dehydrated native BC films. We presume that the decellularization treatment resulted in compaction of the BC structure, that in turn, increased the likelihood and the net strength of interactions between macromolecules.
- The calcium phosphate modification slightly (by 14%) increased the stiffness of the samples. The mechanical properties of the Ag-modified group were found very similar to those of the unmodified “native” samples.

## Figures and Tables

**Table 1:** Mechanical properties of dehydrated bacterial cellulose (BC) samples.

Material	h, mm	Young’s Modulus (MPa)	Tensile Strength σ_max_, (MPa)	Elongation at Break (ε_max_) (%)
* Office paper (80 g/m^2^)	0.07 ± 0.01	302 ± 20.9	12.5 ± 0.9	6.0 ± 0.4
Crude dry BC	0.12 ± 0.02	180.3 ± 10.6	11.6 ± 0.8	8.2 ± 0.6
Decellularized dry BC	0.07 ± 0.01	428.0 ± 24.1	26.0 ± 1.6	7.5 ± 0.4
Decellularized dry BC with calcium phosphate	0.07 ± 0.01	491.8 ± 23.8	31.3 ± 2.2	6.1 ± 0.3
Decellularized dry BC with colloid silver	0.07 ± 0.01	432.8 ± 26.2	24.1 ± 2.8	8.0 ± 0.4

* shown for reference.

**Table 2:** Mechanical properties of wet (fresh and rehydrated) BC samples.

Material	h, mm	Young’s Modulus (MPa)	Tensile Strength σ_max_, (MPa)	Elongation at Break (ε_max_) (%)
Crude fresh BC	0.90 ± 0.05	3.1 ± 0.5	1.2 ± 0.2	46.2 ± 3.5
Decellularized wet BC	0.90 ± 0.05	1.5 ± 0.3	0.28 ± 0.02	49.0 ± 3.6
Crude rehydrated BC	0.13 ± 0.01	127.2 ± 13.9	25.1 ± 1.8	21.1 ± 0.9
Decellularized rehydrated BC	0.08 ± 0.01	250.4 ± 20.1	31.4 ± 2.3	18.5 ± 0.8
